# Immunogenicity of Intensively Decellularized Equine Carotid Arteries Is Conferred by the Extracellular Matrix Protein Collagen Type VI

**DOI:** 10.1371/journal.pone.0105964

**Published:** 2014-08-26

**Authors:** Ulrike Boeer, Falk F. R. Buettner, Melanie Klingenberg, Georgios C. Antonopoulos, Heiko Meyer, Axel Haverich, Mathias Wilhelmi

**Affiliations:** 1 GMP- model laboratory for tissue engineering, Hannover, Germany; 2 Division for Cardiac-, Thoracic-, Transplantation- and Vascular Surgery, Hannover Medical School, Hannover, Germany; 3 Department of Cellular Chemistry, Hannover Medical School, Hannover, Germany; 4 Biomedical Optics Department, Laser Zentrum Hannover e.V., Hannover, Germany; Casey Eye Institute, United States of America

## Abstract

The limited biocompatibility of decellularized scaffolds is an ongoing challenge in tissue engineering. Here, we demonstrate the residual immunogenicity of an extensively decellularized equine carotid artery (dEAC_intens_) and identify the involved immunogenic components. EAC were submitted to an elaborated intensified decellularization protocol with SDS/sodium desoxycholate for 72 h using increased processing volumes (dEAC_intens_), and compared to dEAC_ord_ prepared by an ordinary protocol (40 h, normal volumes). Matrix integrity was checked via correlative volumetric visualization which revealed only minor structural changes in the arterial wall. In dEAC_intens,_ a substantial depletion of cellular components was obvious for smooth muscle actin (100%), MHC I complexes (97.8%), alphaGal epitops (98.4% and 91.3%) and for DNA (final concentration of 0.34±0.16 ng/mg tissue). However, dEAC_intens_ still evoked antibody formation in mice after immunization with dEAC_intens_ extracts, although to a lower extent than dEAC_ord_. Mouse plasma antibodies recognized a 140 kDa band which was revealed to contain collagen VI alpha1 and alpha2 chains via mass spectrometry of both 2D electrophoretically separated and immunoprecipitated proteins. Thus, even the complete removal of cellular proteins did not yield non-immunogenic dEAC as the extracellular matrix still conferred immunogenicity by collagen VI. However, as lower antibody levels were achieved by the intensified decellularization protocol, this seems to be a promising basis for further development.

## Introduction

Over the past decade, the decellularization of native tissues has become a common and broadly applied tool for the generation of xenogeneic scaffolds used in tissue replacement therapy [1]. Various decellularized matrices of different origin are reported to be well tolerated in terms of biocompatibility and immune reactions. However, there have also been reports of local inflammatory processes that lead to degenerative changes and tissue calcification, finally resulting in compromised graft functionality [2]–[4]. Thus, the question is still open as to whether components of decellularized xenogeneic scaffolds may evoke adverse immune reactions and to what extent these occur.

Decellularized vascular scaffolds are a promising alternative to alloplastic prostheses in vascular surgery as the latter are prone to thrombotic events and infections. In particular, vascular access for haemodialysis requires prostheses which have a reduced risk for graft infections, low thrombogenicity, and with the potential to withstand frequent graft punctures. The decellularized equine *Arteria carotis* (dEAC) is considered to fulfil these requirements in terms of the regenerative potential of scaffolds per se, but also specifically due to its size and the absence of multiple outlets, which can complicate the use of the bovine *A. thoracica*, which has approximately the same diameter. Moreover, equine tissue has an added advantage in terms of market authorization due to the absence of transmissable spongiform encephalopathy in this species.

We recently reported on a detailed matrix characterization of vascular grafts obtained by the decellularization of native equine carotid arteries using detergents and endonuclease [5]. This protocol is defined as the ordinary protocol in the current study. In that study, we identified not only an array of residual proteins including αGal residues and MHC I-complexes, but also demonstrated that extracts of these matrices were able to induce antibody formation in mice. Moreover, dEAC_ord_ implanted as arterio-venous shunts in a sheep model evoked sustained adverse immune reactions within a period of 14. These reactions included local adverse tissue reactions such as inflammation and fibrosis, as well as adaptive immune responses i.e., plasma antibody formation and lymphocyte activation [6]. Although most of these grafts were partially repopulated by endothelial and smooth muscle cells and remained patent over the observation time of 14 weeks, all of these tissue processes and immune responses are likely to compromise graft functionality over time. Thus, an improvement of the decellularization process to reduce or even avoid the risk of adverse tissue responses is urgently required.

The term “decellularization” implies the complete removal of cellular components from an extracellular matrix [7]. However, as we demonstrated by a proteomic approach, current strategies are far from achieving a complete removal of cells and cellular components [5]. In particular, DNA [8], αGal epitopes [9] and proteins such as MHC I-complexes [10] are considered to be responsible for the limited biocompatibility of a scaffold. Consequently, the removal of these molecules seems to be pivotal and is, in general, assumed to be the main criterion to predict whether a scaffold is immunologically inert. In contrast, the extracellular matrix displays high levels of similarities across the species and is therefore considered to be non-immunogenic [11]. However, as even small structural differences are sufficient to identify the matrix as of foreign origin, it cannot be ruled out that components of the extracellular matrix could also contribute to scaffold immunogenicity. Thus, in order to assess the actual immunogenic potential of a scaffold, an in vivo evaluation is necessary.

In the present study, we aimed to evaluate decellularized equine carotid arteries which had been generated by an intensified decellularization protocol in particular with regard to (i) the efficacy of depletion of residual cellular components/molecules; (ii) the immunogenicity of the resulting scaffolds by an in vivo mouse model and (iii) and the identification of immunogenic proteins by a proteomic approach. We were able to show that the intensified decellularization removed virtually all cellular components. Although immunogenicity was reduced, it was not entirely eliminated and was shown to be directed against an extracellular matrix component.

## Materials and Methods

### 1. Decellularization protocols

**Figure 1:**
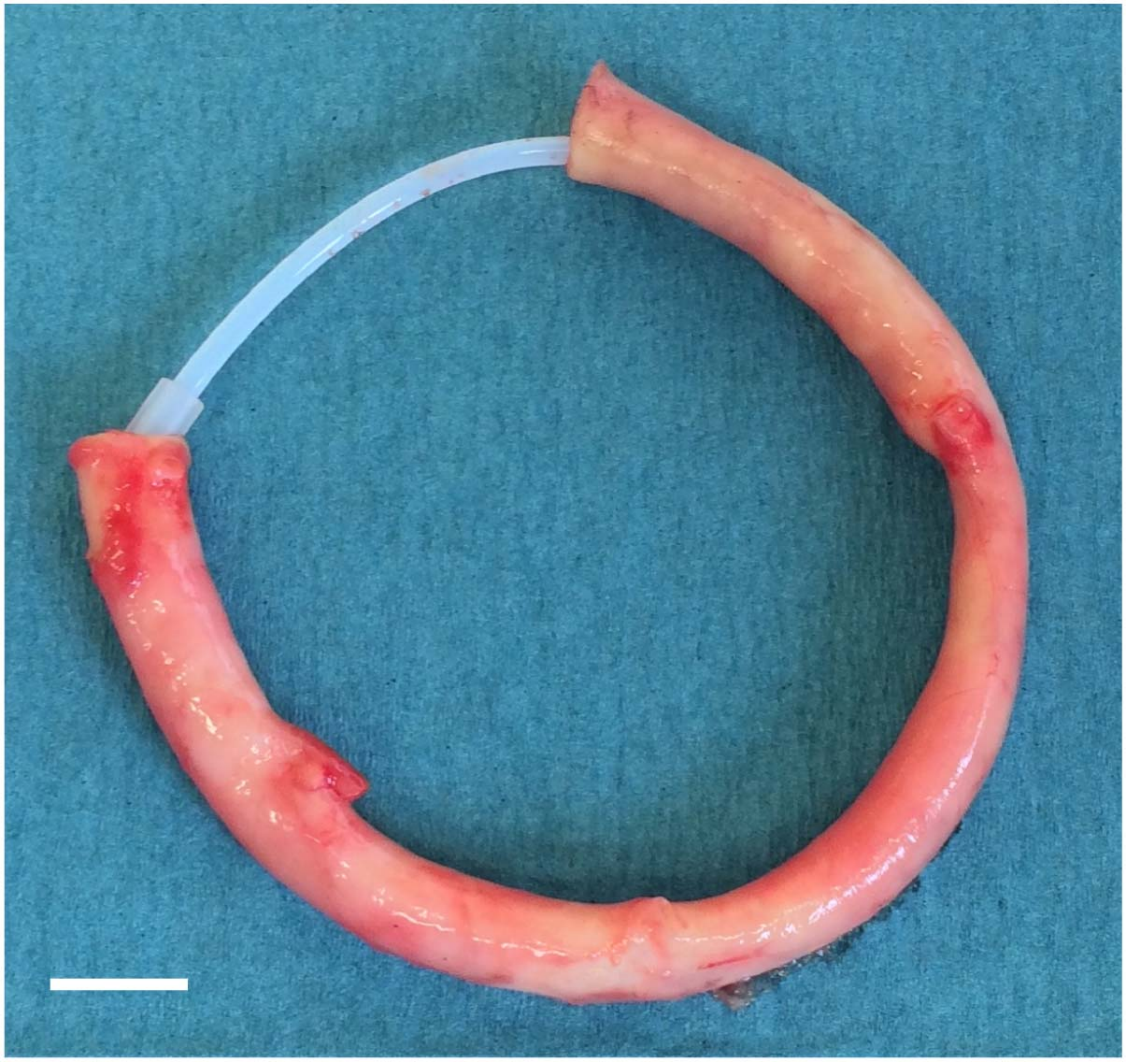
Intensified decellularization of equine *Arteria carotis*. Native carotid artery threaded onto a Teflon tube prior to decellularization with 300 mL of detergent solution for 72 h. Scale bar: 1 cm.

Equine Carotid arteries (equine *Arteria Carotis*, EAC) were obtained from a local slaughter house under semi-sterile conditions and stored in cold 0.9% NaCl+1% penicillin/streptomycin until further processing. Adjacent tissue was removed carefully and carotids were disinfected with 70% ethanol for 20 min and washed with 0.9% NaCl. Then, two different decellularization processes were initiated. For ordinary decellularization, EAC pieces of 10 cm length were transferred to 250 mL bottles containing 100 mL of decellularization solution (0.5% SDS and 0.5% sodium deoxycholate) and shaken for 40 h. After intense washing with distilled water (3 cycles with 100 mL for 15 min) and 0.9% NaCl (8 cycles with 100 mL for 12 h) EAC were treated with 75 U/mL endonuclease (Merck, Darmstadt, Germany) in 100 mL for 4 h at 37°C. Finally, EAC were washed with 100 mL 0.9% NaCl (two cycles of 15 min and two for 12 h). EAC decellularized by the ordinary protocol were termed dEAC_ord_. For intensified decellularization, EAC pieces of 10 cm in length were threaded onto rings of Teflon tubes to prevent from the arteries from collapsing arteries and to improve purging (Fig. 1). Rings with the carotids were transferred to 500 mL bottles containing 300 mL of decellularization solution (see above) and shaken for 72 h. The next steps were performed as described above, but each with 300 mL solution. EAC decellularized by the intensified protocol were termed dEAC_intens_. All changes of solutions were performed under sterile conditions. Both protocols were run throughout under vigorous shaking on an orbital shaker with an orbit of 30 mm (KS 501 D, IKA, Staufen, Germany).

### 2. Sterility testing of decellularized (d)EAC

The sterility was checked by the incubation of pieces from each end of the graft in caso media (Roth, Karlsruhe, Germany). Moreover, the wash solution of the last washing step was mixed 1∶6 with 6-fold concentrated caso media. Grafts were considered sterile if no bacterial growth occurred in the respective media after 14 days at 37°C. Sterile dEAC were stored in 0.9% NaCl+1% penicillin/streptomycin at 4°C.

### 3. DNA content

Residual DNA content within dEAC following the two decellularization protocols was quantified by Quant-iT Picogreen Assay (Invitrogen, Darmstadt, Germany) and according to the manufacturer’s instructions. Briefly, tissue samples of 25 mg were digested by proteinase K, DNA was isolated by affinity columns (DNeasy Blood and Tissue Kit, Qiagen, Hilden, Germany), lyophilized and, after addition of the fluorescent dye, the DNA content was quantified by a microtiter plate fluorescence reader (DTX800, Beckmann-Coulter, Krefeld, Germany) at an excitation of 485 nm and measurement at 520 nm.

### 4. Volumetric visualization

**Figure 2:**
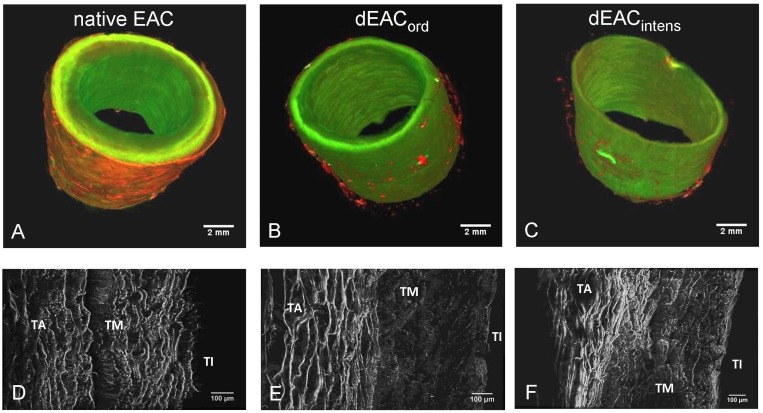
Correlative volumetric visualization of the carotid wall for native EAC, dEAC_ord_ and dEAC_intens_ by Scanning Laser Optical Tomography (SLOT). SLOT (A–C) was performed in transmission mode displaying autofluorescence at 532 nm on tissue pieces of 1.5 cm in length from the indicated tissues and by Multi Photon Microscopy (D–F) with maximum intensity projections of axial cross sections of the indicated tissues representing the autofluorescence at 800 nm excitation wavelength. TA: tunica adventitia; TM: tunica media; TI: tunica intima.

Correlative volumetric visualization of the carotid wall for native carotids (nEAC), dEAC_ord_ and dEAC_intens_ was performed using multi photon microscopy (MPM) (TriM Scope II, LaVision Biotec, Bielefeld, Germany) and scanning laser optical tomography (SLOT, custom setup, Laser Zentrum Hannover e.V, Germany). For MPM, nEAC, dEAC_ord_ and dEAC_intens_ were cut along their longitudinal axis. The samples were fixed in a petri dish using quick glue and covered with 1x phosphate buffered saline (PBS) to gain the index match for the water immersion objective (W Plan-Apochromat, 20x/1.0, Zeiss, Jena, Germany). Illumination was generated using a fs-laser (Chameleon Ultra II, Coherent, USA) operating at 830 nm, 140 fs pulse length with a repetition rate of 80 MHz. Imaging of the carotid wall cross section was performed applying the mosaicking mode using ImSpector Pro software (LaVision BioTec, Bielefeld, Germany) with a total field of view of 1164×819 µm for 40 h, 1443×834 µm for 72 h and 1078×849 µm for the native specimen. Fig. 2 represents the maximum intensity projection of multiphoton images generated with the open source software ImageJ (http://imagej.nih.gov/ij/, USA).

Additionally, specimens for SLOT imaging were prepared in parallel from the same samples. 1.5 cm pieces of native and decellularized EAC were dehydrated by increasing ethanol concentrations and then cleared using a 1∶2 BABB (benzylalcohol benzyl benzoate) solution resulting in a refractive index of n = 1.5585. Samples were scanned in transmission mode and for autofluorescence by exposition with a wavelength of 532 nm.

### 5. Homogenization of EAC

400 mg native EAC (nEAC), dEAC_ord_ and dEAC_intens_ were homogenized using a bead mill followed by ultrasonification. Homogenates were extracted for 1 h at room temperature by 1 mL detergent solution containing 5% SDS, 5% sodium deoxycholate, 1 mM EDTA and 10% (v/v) protease inhibitor cocktail (Sigma-Aldrich, Steinheim, Germany; cat. no. P2714) in PBS and centrifuged afterwards. Aqueous extracts were obtained with PBS only. Protein content was determined by Pierce BCA assay (Thermo fisher Scientific, Dreieich, Germany).

### 6. Western blot analyses

For western blot analyses, equal amounts (10–30 µg protein) of extracts from dEAC_ord_ and dEAC_intens_ were separated by 10–12% SDS-PAGE and transferred to PVDF membranes by tank blotting over night at 4°C. Membranes were probed by specific antibodies for smooth muscle actin (SMA, Acris Antibodies, Herford, Germany, DM001; 1∶10.000), equine major histocompatibility complex (MHC) class I (AbD Serotec, Oxford, UK; 1∶10), plasma from immunized mice (1∶2000) and peroxidase-coupled anti mouse secondary antibodies. For αGal epitope detection, the blot was incubated with peroxidase-coupled *Bandeiraea simplicifolia* isolectin B4 (Sigma-Aldrich, Steinheim, Germany, L5391; 1 µg/mL) in TBS containing 1 mM CaCl_2_, 1 mM MgCl_2_, 1 mM MnCl_2_ and 0.5% Tween-20. Bound antibodies and isolectin were visualized with ECL plus system (GE Healthcare, Munich, Germany) and all bands were quantified densitometrically using the software QuantityOne (Biorad, Munich, Germany). The degree of depletion (%) was calculated from the bands by the formula:

dd (%) = (density dEAC_intens_ *protein content dEAC_intens_/density dEAC_ord_*protein content dEAC_ord_)*100.

### 7. Immunization of NMRI mice

Five female NMRI mice were immunized with aqueous extracts from dEAC_intens_ coupled to Gerbu adjuvant (Gerbu Biotechnik GmbH, Weiblingen, Germany). At day 1, mice received 30–35 µg protein and at day 4, 8, 11 and 14 15–20 µg protein per day by i.p. injection. Mice were sacrificed at day 17 and lithium heparin plasma was collected retroorbitally. Controls (n = 3) received Gerbu adjuvant in solvent. Plasma from the immunization with extracts from dEAC_intens_ (plasma_intens_) were compared to plasma obtained by immunization with extracts from dEAC_ord_ which have been described before (plasma_ord_; [5]).

All experimental procedures were carried out in strict compliance with the “Guide for the Care and Use of Laboratory Animals” of the National Institutes of Health (Washington DC: National Academy Press; 1996 www.nap.edu/catalog/5140.html) and were approved by the Committee on the Animal Care and Use of the local institution and state (“Landesamt für Verbraucherschutz und Lebensmittelsicherheit”, Oldenburg, Germany).

All efforts were made to minimize suffering of the animals.

### 8. 2-dimensional (2D) gel electrophoresis

2D electrophoresis was performed essentially as described previously [12]. In brief, Immobiline DryStrips (24 cm, 3–11 NL, Amersham, Freiburg, Germany) were rehydrated with 2D electrophoresis buffer (7 M urea (Roth, Karlsruhe, Germany), 2 M thiourea (Sigma-Aldrich, Steinheim, Germany), 4% [w/v] CHAPS (Roth) 1% [v/v] IPG buffer 3–11 NL) supplemented with 1.2% [v/v] DeStreak reagent (Amersham). Extracts of dEAC_intens_ (2×400 µg) were precipitated with acetone and pellets were dissolved in 2D electrophoresis buffer supplemented with 1% [w/v] DTT. Isoelectric focusing was performed in an Ettan IPGphor (Amersham) using anodal cup loading. For the second dimension, IPG strips were equilibrated twice with equilibration buffer (100 mM Tris-HCl pH 8.0, 6 M urea, 30% [v/v] glycerol (Roth), 2% [w/v] SDS (Serva, Heidelberg, Germany) supplemented with 0.5% [w/v] DTT for the first equilibration step and 4.5% [w/v] iodoacetamide (Sigma-Aldrich) for the second step. The second dimension was performed with 12.5% polyacrylamide gels applying the Ettan DALTsix electrophoresis system (Amersham). Two identical experiments were performed and the gels were either stained with silver [13] or subjected to western blotting.

### 9. Immunoprecipitation (IP)

dEAC_ord_ and dEAC_intens_ were homogenized as described above and extracted in IP buffer containing 150 mM NaCl, 25 mM Tris/HCl pH 8.0, 1 mM EDTA, 0.5% sodium desoxycholate and 0.5% Triton X-100 and protease inhibitors. To establish the IP protocol, 300 µg of dEAC_ord_ extracts were incubated with 3 µl plasma from immunized mice (plasma_ord_) overnight at 4°C in a volume of 1.5 mL under gentle agitation. 150 µl 10% (v/v) Protein A sepharose (Sigma-Aldrich, Steinheim, Germany) were added for 2 h at 4°C under gentle agitation. Immunoprecipitated proteins were washed 5 times with IP buffer, dissolved in SDS sample buffer and separated on 8% SDS-PAGE. Precipitated proteins were electrotransferred to PVDF membrane and stained with plasma_ord,_ as described above. For mass spectrometry, the IP of dEAC_ord_ and dEAC_intens_ was up-scaled to 3×1 mg total protein incubated with 15 µl plasma from the respectively immunized mice. Immunoprecipitated proteins per assay were separated on three lanes by SDS-PAGE using 8% gels, subsequently coomassie-stained and submitted to mass spectrometry.

### 10. Protein identification by liquid chromatography-mass spectrometry/mass spectrometry (LC-MS/MS)

Spots of the 2D electrophoresis (sample S1) or protein bands from the immunoprecipitation (samples S2–S9) were excised, cut into small pieces and subjected to in-gel digestion with trypsin according to standard procedures [14]. In brief, the gel pieces were dehydrated with acetonitrile (ACN, Merck, NJ, USA); proteins in the gel were subsequently reduced with 10 mM DTT (Sigma) in 100 mM ammonium bicarbonate buffer (AmBic) and carbamidomethylated with 100 mM iodoacetamide (Sigma-Aldrich, Steinheim, Germany) in 100 mM AmBic. After dehydration with ACN, rehydration with 100 mM AmBic and dehydration with ACN, the gel pieces were rehydrated with 50 mM AmBic containing 20 ng/µl sequencing-grade trypsin (Promega, Mannheim, Germany) and incubated for 16 h at 37°C. The peptides were extracted with ACN and dried in a vacuum centrifuge. The dried peptides were dissolved in 15 µl of 2% ACN and 0.1% formic acid and the supernatant was subjected to LC-MS/MS analysis. Reverse phase chromatography using acetonitrile as an eluent was performed on a Waters nanoACQUITY UPLC (Milford, USA) device equipped with an analytical column (Waters, BEH130 C18, 100 µm×100 mm, 1.7 µm particle size) coupled online to an ESI Q-TOF Ultima (Waters). Spectra were recorded in positive reflection mode and peptides were automatically subjected to fragmentation. Protein identification was performed applying the ProteinLynx Global Server software V2.1 (Waters) by searching in a horse database (uniprot-equus+caballus.fasta, downloaded on 13.09.2013 from Protein Knowledgebase UniProtKB [http://uniprot.org/]). Carbamidomethylation was set as fixed modification and oxidation of methionine as variable modification. Up to one missed cleavage was allowed. Peptide tolerance was set to 100 ppm and fragment tolerance to 0.1 Da and the validation filter was selected in the ProteinLynx Global Server software.

### 11. Statistics

Statistical calculations were done using Graphpad Prism 5.04 (GraphPad Software, San Diego, California) statistic functions. The mean and standard error of the mean (SEM) were calculated for all values obtained in this study. Gaussian normal distribution was tested by d’Agostino & Pearson’s omnibus normality test. Two group-comparison for non-parametric data was done by the Mann Whitney U test and by Student’s t-test for normally distributed values. Multiple comparison between groups was performed by two-way ANOVA and Bonferroni post test. Differences were considered significant at p<0.05. Significance levels were given as follows:*p<0.05; **p<0.01 and §p<0.0001.

## Results

### 1. Intensified decellularization of dEAC

The existing decellularization protocol described recently [5] was changed with regard to the decellularization time and the volumes used to increase the efficacy of removing cellular components. Moreover, a Teflon scaffold was introduced to improve the purging of the carotid artery by the respective solution (Fig. 1). For a first assessment of the decellularization efficacy, the residual DNA content was quantified. As previously shown, dEAC_ord_ revealed a very low residual DNA content of 0.75±0.44 ng/mg tissue. The intensified decellularization protocol further decreased the DNA content to 0.34±0.16 ng/mg which was significantly less (p<0.05) and therefore was assumed to be more effective than the ordinary protocol.

### 2. Effects of decellularization on arterial wall structure

The effects of the ordinary and the intensified decellularization procedure on the structure of the extracellular matrix of the arterial wall were investigated by scanning laser optical tomography (SLOT) and multi-photon microscopy (MPM; Fig. 2). SLOT revealed a loss in wall thickness using both decellularization protocols, which affected predominantly the outer layer (tunica adventitia) and seemed to be slightly more pronounced for the dEAC_intens_ (Fig. 2A–C). MPM elucidated this observation by showing a shrinkage of elastine fibres in the tunica adventitia and a substantial loss and shrinkage of fibres in the tunica media. As before, these findings seemed to be somewhat more strongly evident in dEAC_intens_ (Fig. 2D–F).

### 3. Western blotting of residual proteins

**Figure 3:**
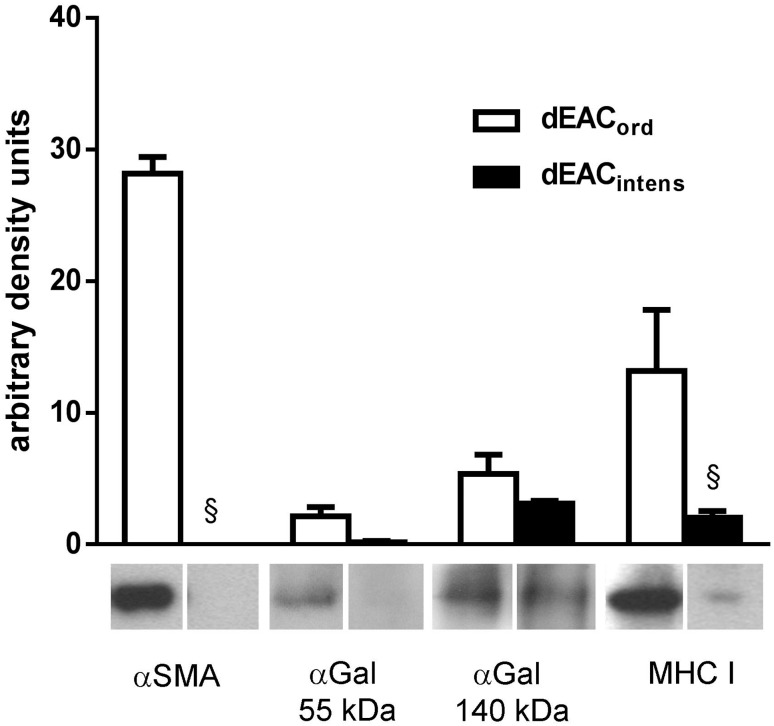
Western blot analyses of extracts of dEAC_ord_ and dEAC_intens_. 30 µg of protein were separated on SDS-PAGE, transferred to PVDF membrane and probed by the indicated antibodies or peroxidase-linked *Bandeiraea simplicifolia* isolectin B4 for αGal detection. Bands of three different carotids were quantified densitometrically and shown as the means ± SEM. §: p<0.0001 by two-way ANOVA and Bonferroni post test.

To evaluate the decellularization efficacy beyond the DNA content, cellular proteins which are supposed to contribute to immunogenicity of a matrix were analyzed in dEAC_ord_ and dEAC_intens_. Alpha smooth muscle actin (αSMA) as integral part of the cytoskeleton, αGal residues and MHC I-complexes, representing the most immunogenic molecules, were detected by western blots in matrix extracts and quantified by densitometry. In recent work conducted by our group, it was shown that, using the ordinary protocol, αSMA in dEAC_ord_ was depleted to 13.8%; MHC I to 14.8% and αGal to 15.1% compared to the native EAC [5]. Using the intensified protocol, this depletion was considerably increased. On western blots of dEAC_intens,_ virtually no αSMA could be detected, indicating that it had been completely eliminated (0% of dEAC_ord_, p<0.0001; Fig. 3). MHC I was detected as faint band on the blots indicating a highly significant depletion of 15.5% compared to dEAC_ord_ (p<0.0001). Moreover, staining of αGal residues on blots of dEAC_intens_ extracts revealed two distinct bands at 55 kDa and of 140 kDa, which were reduced to 10.7% or to 57.7% of dEAC_ord_ respectively (marginally significant, p<0.07 and p<0.09). Related to the protein content in native EAC [5], total depletion could be calculated to 100% for αSMA, 97.8% for MHC-1 and 98.4% and 91.3% for αGal residues.

### 4. Immunogenicity of dEAC_intens_ and identification of immunogenic proteins

**Figure 4:**
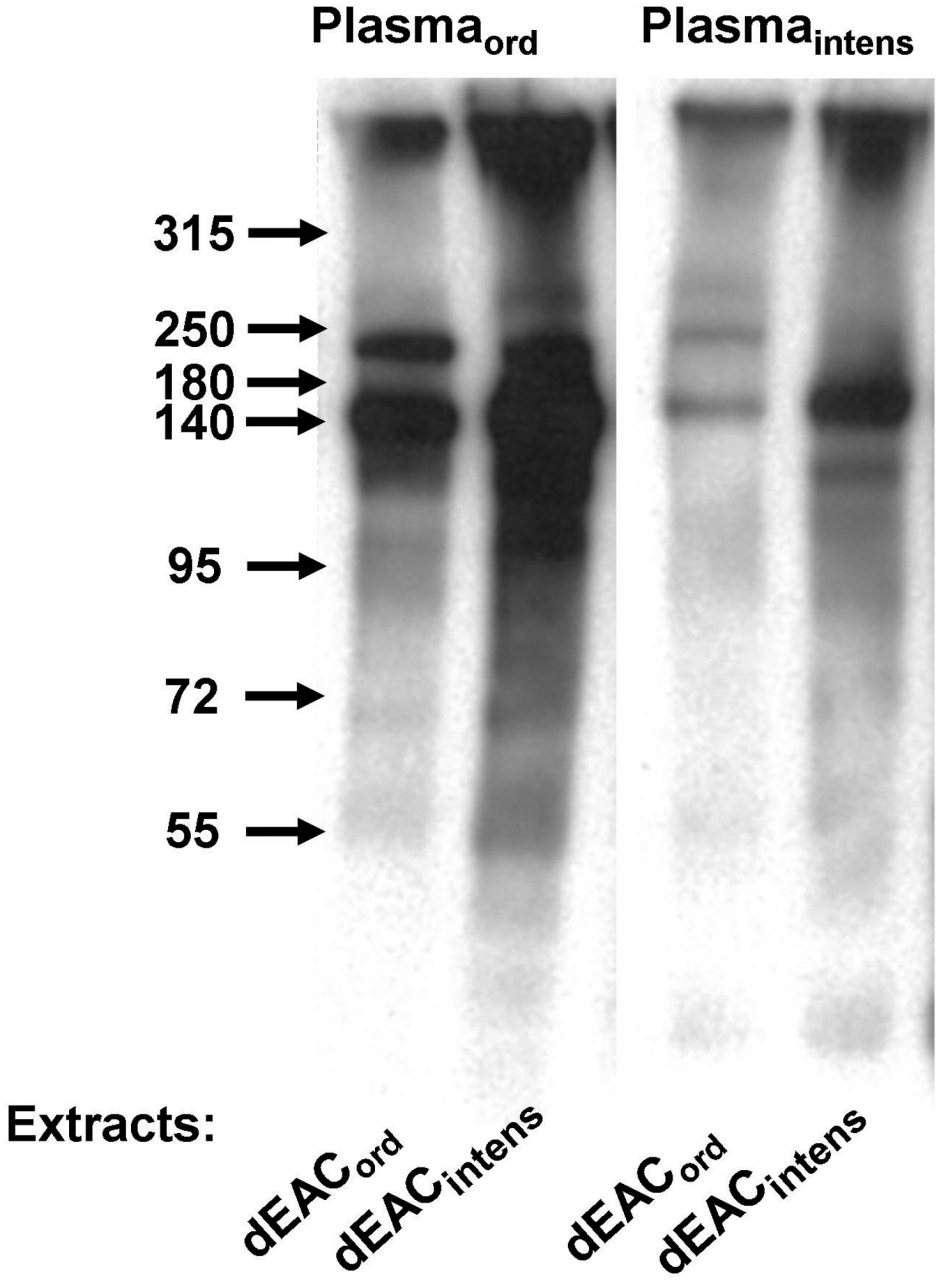
Detection of specific antibodies in mouse plasma after immunization with dEAC_ord_ (plasma_ord_) and dEAC_intens_ (plasma_intens_). NMRI mice were injected with aqueous extracts of dEAC_ord_ or dEAC_intens_ over 17 days and plasma was probed as a primary antibody on 30 µg of extracts of dEAC_ord_ and dEAC_intens_ seperated by SDS-PAGE and transferred to PVDF membrane. Bound antibodies were visualized with anti-mouse peroxidase-labeled secondary antibodies and the enhanced chemiluminescence system. A typical blot of three independent similar experiments is shown.

To evaluate whether dEAC_intens_ were still immunogenic, we immunized mice with aqueous extracts of the matrices and analyzed the collected murine plasma_intens_ in western blot analyses. Fig. 4 shows a typical western blot with extracts of dEAC_ord_ and dEAC_intens_ separated by SDS-PAGE and transferred to PVDF-membrane which was probed by the plasma of mice immunized with dEAC_ord_ extracts (plasma_ord_) or immunized with dEAC_intens_ extracts (plasma_intens_).

**Figure 5:**
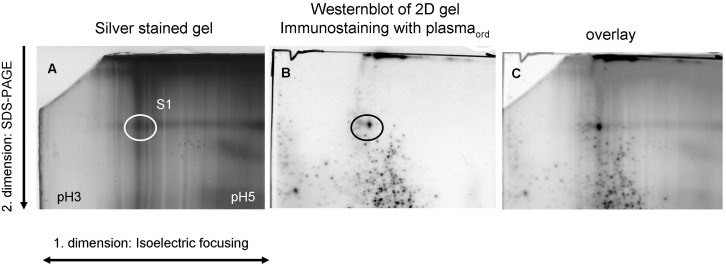
2D electrophoresis of dEAC_intens_. 2×400 µg protein were submitted to isoelectric focusing (first dimension) and SDS-PAGE (second dimension). One gel was silver stained (A), the other was blotted to PVDF membrane and probed with plasma from mice immunized with dEAC_ord_ (plasma_ord_) and anti-mouse peroxidase secondary antibody. One spot stained (black circle) was colocalized (C) with silver stained proteins in A (white circle). The spot was excised from the silver stained gel and submitted to mass spectrometry as sample S1.

**Figure 6:**
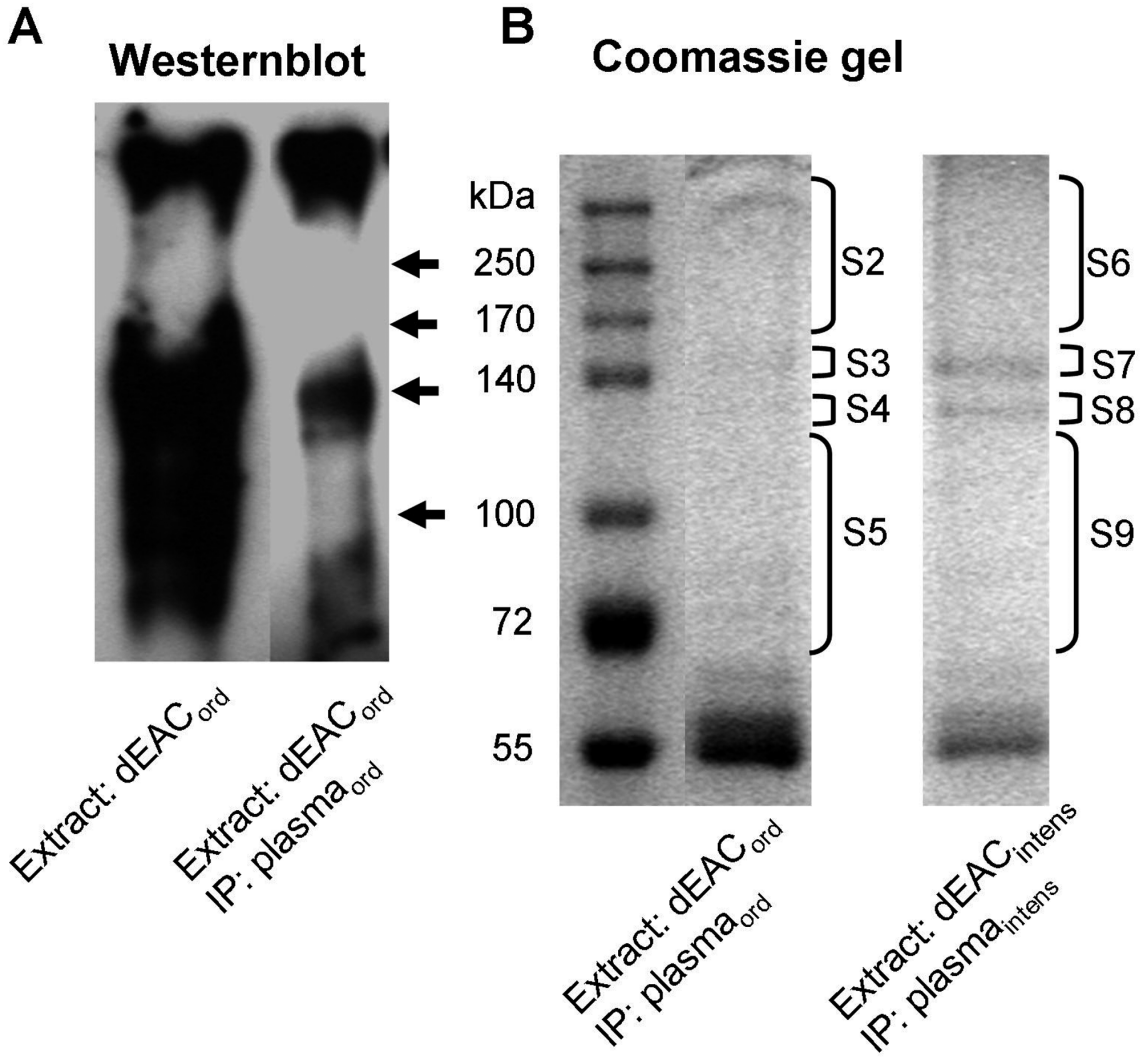
Immunoprecipitation of immunogenic proteins. 1 mg of dEAC_ord_ and dEAC_intens_ extracts were incubated with plasma from mice immunized with dEAC_ord_ (plasma_ord_) and dEAC_intens_ (plasma_intens_) overnight, immune complexes were precipitated with protein A agarose, separated by SDS-PAGE and coomassie-stained. Bands form the gel were excised as indicated by the brackets and submitted as samples S2–S9 to mass spectrometry.

The four lanes showed similar patterns of stained bands with the most prominent one at 140 kDa and one of less intensity at 240 kDa, which was stained by both plasmas. In dEAC_intens_ extracts, a further band appeared between 140 and 95 kDa which was also stained by both plasmas. As the 140 kDa band appeared to display the most prominent protein in dEAC_ord_ and the only immunogenic protein remaining in dEAC_intens_, we aimed to identify this band using a proteomic approach. For this purpose, a two-dimensional (2D) electrophoretic separation of the residual proteins of dEAC was performed. According to Fig. 4, the strongest immunostainings were obtained by using dEAC_intens_ extracts and plasma_ord_. Therefore, this combination was chosen for the 2D electrophoresis approach. Two identical gels were prepared: one was silver stained (Fig. 5a) and the other was subjected to western blotting and immunoprobed with plasma_ord_ (5b). Here, a single dot was stained which was located in the acid region of the first dimension at higher molecular weights. This dot was colocalized (Fig. 5c) with an assembly of silver stained proteins from where a spot was excised (sample S1) and submitted to mass spectrometry. We were able to identify two proteins, both belonging to the same extracellular matrix molecule: Equine collagen type VI α1 and α2 chain (table 1), indicating that the extracellular matrix components and not cellular proteins conferred immunogenicity. To confirm this unexpected finding by a second approach, we performed an immunoprecipitation with the immunoplasma and the respective tissue extracts. Fig. 6A shows a western blot of precipitated proteins which are stained in the 140 kDa region as the blotted extract, confirming that the immunoprecipitation had worked. Up-scaling of the assay resulted in higher amounts of precipitated proteins which could be stained by coomassie (6B). For the dEAC_intens_ extracts precipitated with plasma_intens,_ a clear band at 140 kDa and a weaker one at about 120 kDa was visible. For the dEAC_ord_ extracts precipitated by plasma_ord,_ only faint bands at the same molecular weights were detected. Bands were excised (samples S2–S9) and proteins were analyzed by mass spectrometry. As before with the 2D electrophoresis, the proteins in the 140 kDa band of the dEAC_intens_ sample (sample S7) were revealed to be collagen type VI, α1 and α2 chain (table 1). For the dEAC_ord_ no proteins could be identified which most likely due to the tiny amounts of precipitated proteins was falling below the detection limit of this method.

**Table 1:** Immunogenic Proteins identified by mass spectrometry.

protein identification^a^					mass spectrometry^b^			
sample #^c^		UniProtKBprotein name	UniProtKBaccession number	theoretical mW[kDa]/pI	probability [%]	matched peptides	coverage [%]	mean error[ppm]^d^
Fig. 5	S1	**COL6A2**	F7CGV8	105/5.9	100	4	3.7	18.7
Fig. 6	S7	**COL6A2**	F7CGV8	105/5.9	100	8	9.1	9.7
		**COL6A1**	F6UW03	109/5.1	100	6	7.1	8.9

From gels containing 2D electrophoresis, separated (Fig. 5) or immunoprecipitated (Fig. 6) proteins spots or bands were excised, trypsin-digested and submitted to mass spectrometry.

a Processed mass spectra were searched for in the “equus caballus” database downloaded from UniProtKB.

b Mass spectra were processed with ProteinLynx global server software V2.1 (Waters).

c Sample number as indicated on the respective figure.

d Mean error of all peptides measured.

Thus, two different approaches revealed collagen VI α1 and α2 chains as immunogenic proteins of decellularized EAC inducing antibody responses in the xenogeneic model chosen.

**Figure 7:**
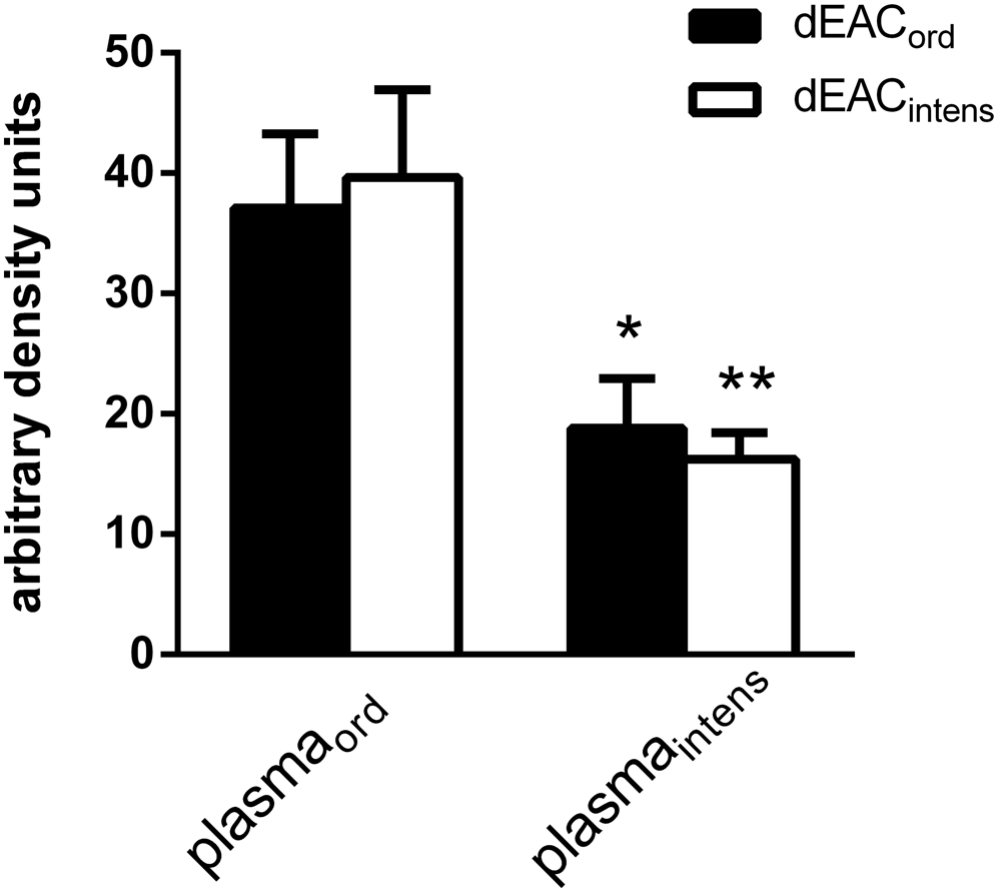
Quantification of specific antibodies in mouse plasma after immunization with dEAC_ord_ (plasma_ord_) and dEAC_intens_ (plasma_intens_). On the western blots as described in Fig. 4, the 140 and the 240 kDa bands of the dEAC_ord_ extracts (n = 7) and of the dEAC_intens_ extracts (n = 9) probed with the respective plasma were quantified densitometrically. Shown are th means ± SEM. *: p<0.05 and **: p<0.01 between plasma_ord_ and plasma_intens_ by Student’s t-test (plasma_intens_) or Mann-Whitney U test (plasma_ord_).

In Fig. 4, the overall intensity of the bands stained by plasma_ord_ seemed to be higher; therefore the 140 kDa and the 240 kDa bands were quantified densitometrically. Fig. 7 shows a clearly reduced staining by plasma_intens_ for both extracts (41.7%, p<0.05 for the dEAC_ord_ extract and 40.9%, p<0.01 for the dEAC_intens_ extract) whereas there was no difference between the extracts themselves. This indicates a reduced amount of antibodies formed after immunization with dEAC_intens_ extracts in mice but a similar specificity of these antibodies to predominantly collagen type VI.

## Discussion

Although the development of decellularized xenogeneic scaffolds has undergone continuous development over the last decade, there is still an ongoing debate as to what extent matrices contribute to immunogenicity and how safe they are when used for life-long replacement therapy.

Most recently, a comprehensive review on scaffold immunogenicity highlighted the necessity to reduce immunogenic components from tissues, described the limitations of established decellularization protocols and of the evaluation of acellularity, and suggested more sophisticated procedures to remove antigens [15]. In the context of this review, our study adds valuable information concerning the benefits and the limitations of an intensified detergent-based decellularization procedure of equine carotid arteries. Moreover, we suggest that the immunogenic principle inducing antigen response in completely acellular scaffolds is related not to cellular proteins but to structural component of the extracellular matrix, namely collagen VI.

In order to optimize the decellularization for equine carotids and thus to reduce their immunogenicity, changes to the previous standard protocol were made regarding the decellularization time and the intensity of purging to remove solubilized cellular components.

The most obvious and expected outcome of these changes was its efficacy in terms of depletion of immunogenic proteins. Intensified decellularization resulted in a higher degree of depletion of DNA and cellular proteins than our ordinary protocol [5]. In our previous study, a complete overview of all proteins in dEAC_ord_ was given comprising cytosolic, structural, membrane and nucleic proteins. Here, we assume that the depletion of cytosolic, cytoskeleton and membrane proteins using the intensified protocol was almost complete. αSMA was completely eliminated (100%) and MHC I-complexes were depleted to 97.8%; therefore, we assume that these molecules can be excluded as contributing to the immunogenicity of dEAC_intens_.

αGal as the widely discussed antigen responsible for xenogeneic transplant rejection [16] was detected at two distinct bands, of which the 55 kDa was depleted to 98.4%, whereas the 140 kDa band was depleted to a slightly lesser extent to 91.3%. The lower band most likely indicates a protein which is solubilized by the intensified detergent treatment and thus demonstrates an example for successful antigen removal, as outlined by Wong et al. [17]. However, the 140 kDa band resisted intensified detergent treatment and thus displayed an αGal-glycosylated protein which is assumed to be stably integrated in the extracellular matrix of the scaffold itself.

Interestingly, the immune plasma obtained from mice immunized with dEAC_intens_ stained a single band of the same size, which therefore suggests that it contains a protein which is pivotal for scaffold immunogenicity.

In this study, two different proteomic approaches identified this immunogenic 140 kDa protein as collagen VI α1 and α2 chain. Collagen VI is a component of the extracellular matrix in many connective tissues, but also an integral part of arterial walls [18]. It was first described in extracts of arterial tunica intima [19] and later as part of so-called oxytalan fibres in the tunica media connecting the basal lamina of smooth muscle cells with elastic lamellae [20]. Collagen VI consists of up to 6 chains [21] that are built up by varying numbers of von-Willebrand factor A-like globular domains and contains a triple helix domain which is important for assembly stabilization [21]. Collagen VI monomers are formed by the short α1 and α2 chains (105 kDa) and the longer α3 chains (345 kDa) which can be replaced by α4–6 chains [21]. Collagen VI was identified as a 140 kDa band on SDS-PAGE in 1985 [22] which, however, based on the current knowledge, could not be allocated to the molecular weight of a certain chain or the sum of chains. It is most likely that glycosylation of the α1 and α2 chains results in the apparent molecular weight of 140 kDa, whereas the α3 chain of 345 kDa plus glycosylation probably does not migrate into the gel. As we found strongly immunostained proteins on the upper rim of the gels (Fig. 4), one could speculate that these bands are formed by α3 chain of collagen VI.

The immunogenicity of collagens has been described thoroughly for soluble forms like collagen type I and III, which are used for biomedical devices and soft tissue augmentation. Adverse reactions, such as localized inflammation and allergy induction, have been shown to depend on the donor and recipient species, as well as on the structural part (triple helix or telepeptides) of the molecule and to include humoral and cellular immune reactions [23]. Also, collagen type II is a known inducer of autoimmune responses: When injected into mice, collagen II induced an autoimmune arthritis that resembles rheumatoid arthritis [24]. Moreover, in patients with bullous disorders, serum antibody levels against type III, IV and V collagens were detected [25]. To our knowledge, there is only one study identifying collagen VI as an antigen inducing beef allergy in sensitized individuals [26]. Sera from 29 patients tested in western blots with beef and pork homogenates revealed strong IgE binding to two proteins of 240 and 140 kDa. Mass spectrometry identified these proteins to be laminin γ1 and collagen VI α1 chain. Moreover, it was suggested that the immunogen in these proteins was αGal, as periodate treatment diminished IgE binding. There was, however, no comment on the interesting question as to why exclusively αGal residues on these two proteins were highly immunogenic and not on others. This study is both complementary and contradictory to our results. First, it confirms the immunogenicity of the xenogeneic scaffold protein collagen VI, as we identified exactly the same molecule as causing antibody formation. It also widens the view of the immunogenicity of proteins from long-term exposure, as in the therapeutical implantation of decellularized tissues, to the short-term in repeated exposure by oral ingestion. Moreover, the fact that collagen and laminin were able to induce an immune response highlights the role of highly conserved proteins in this context. However, the notion that the αGal epitope is responsible for the immunogenicity of collagen VI is not supported by our results. Although isolectin B4 staining of a 140 kDa band most likely displays αGal-glycosylated collagen VI, in our experimental setting αGal immunogenicity between two species both expressing α1,3 galactosyl transferase was excluded. It is, however, striking that the same protein that primarily presents αGal residues to the human immune system also confers immunogenicity between species where αGal is irrelevant.

At this point, we cannot explain in detail why specifically collagen VI α1 and α2 chains are immunogenic, despite being conserved to 89% or 93% between horses and mice, respectively (see Fig. S1 and S2, alignments of collagen VI α1 and α2 chains). However, as reviewed by Lucchese [27], even small differences in amino acid sequences can result in conformational changes of the spatial structure of proteins which act in a completely different way to the unchanged proteins, or can also evoke immune responses. Moreover, the immunogenicity of a protein is suggested to be related not only to its chemical or conformational changes in the amino acid structure, but also to the degree of similarity of a mutated peptide to other peptides of the organism. The authors showed that single amino acid changes in peptides causing functional differences, such as antibody recognition or T-cell stimulation, correlated with their number of occurrences in a certain proteome. A comprehensive computerized analysis of all amino acid changes in equine collagen VI α1 and α2 chains for similarities in the mouse proteome would therefore be a suitable tool to predict which part of the molecules displays an enhanced immunogenic risk.

It cannot ruled out that, in addition to collagen VI, further proteins in dEAC_ord_ scaffolds evoke an immune response, but the approaches chosen here were both limited in sensitivity. In western blots, at least two further weak bands were detected which most likely also display extracellular matrix proteins. In particular, the 240 kDa band stained by plasma_ord_ was not detected by either proteomic approach. Takahashi [26] identified the 240 kDa protein inducing beef allergy as Laminin γ1. However, among the residual proteins identified in extracts of dEAC_ord_ listed in [5], no laminin chains were discovered, whereas collagen VI α1, α2 and α3 chains were found. Thus, the nature of this band still remains unknown. However, as this band was almost eliminated in the dEAC_intens_ (Fig. 4), the necessity for its identification seems questionable.

Last but not least, we observed that the overall amount of antibodies towards the matrices was significantly reduced (Fig. 7). Although dEAC_intens_ still contained collagen VI α1 and α2, the intensified protocol seems to produce matrices which are less immunogenic than dEAC_ord,_ since the 240 kDa band disappeared completely and also the 140 kDa band seemed to be less intensive. A possible explanation for this phenomenon is that the prolonged detergent-treatment partly denatures collagen VI and thus attenuates its immunogenic properties. This effect has been shown before for heat or formalin denaturation of ovalbumin, which was less immunogenic when administered in mice [28]. As shown by MPM, the structure of the dEAC_intens_ differed from dEAC_ord_ by displaying shrinked fibres in both the tunica adventitia and tunica media which may include conformational changes of collagen VI. This effect, leading to reduced antibody formation, would be beneficial for the use of these intensively decellularized carotid arteries in humans. However, the implantation of a “naked” scaffold should be considered with care. Although the relevance of antibody formation towards decellularized scaffold is still not clear, it is likely to induce chronic inflammation and graft degeneration. As prolonged and deeper solubilization of matrix compounds is suspected to destroy increasingly the graft substance other strategies should be considered to overcome this problem. In the past, two approaches have successfully improved the biocompatibility in a sheep model. In vitro-recellularization with autologous endothelial cells decreased local inflammatory tissue reactions significantly [29]. The same effect was observed when dEAC_ord_ were coated with CCN1, a matricellular protein [6] which, in addition, also improved the humoral and cellular immune responses. It is likely that this effect is due to the fact that the immunogenic parts of the scaffold were not exposed to the recipient’s immune system due to prior coverage with cells or adaptive proteins. Other strategies have been suggested by Myers et al. for collagen II- induced arthritis. Here, oral desensibilization of mice with recombinant collagen IX [30] or injection of altered peptide ligands as anti-ligands to the T-cell receptor [31] induced the suppression of arthritis. The question of whether these mechanisms could also suppress immunogenicity of collagen VI in decellularized carotids therefore merits further investigation.

## Conclusion

Our results imply that the overall goal of completely eliminating immunogenic proteins from vascular scaffolds by intensified detergent-based decellularization is, in principle, not achievable. As the extracellular matrix proteins collagen VI α1 and α2 chains as integral compounds of the arterial wall confer immunogenicity towards the scaffold, the implantation of xenogeneic tissues which have not undergone further treatment should be carefully considered. However, as prolonged and intensified detergent-treatment significantly attenuated the immune response of equine carotid arteries, this seems to provide a worthwhile basis for the application of coating and recellularization strategies for the successful development of optimized vascular grafts.

## Supporting Information

Figure S1 **Alignment of equine and murine collagen alpha-1 VI amino acid sequences by basic local alignment search tool (BLAST,** http://blast.ncbi.nlm.nih.gov/Blast.cgi **) using the accession numbers XP_001488401.2 (Equus caballus) and NP_034063.1 (Mus musculus).**
(DOC)

Figure S2 **Alignment of equine and murine collagen alpha-2 VI amino acid sequences by basic local alignment search tool (BLAST,** http://blast.ncbi.nlm.nih.gov/Blast.cgi **) using the accession numbers XP_005606307.1 (Equus caballus) and AAH34414 (Mus musculus).**
(DOC)
