# Supplementary material for: Immunogenicity of Intensively Decellularized Equine Carotid Arteries Is Conferred by the Extracellular Matrix Protein Collagen Type VI
Source: PLoS One. 2014 Aug 26;9(8):e105964. doi: 10.1371/journal.pone.0105964 (PMC4144968; doi:10.1371/journal.pone.0105964)
Supplement: Figure S1 — Alignment of equine and murine collagen alpha-1 VI amino acid sequences by basic local alignment search tool (BLAST, http://blast.ncbi.nlm.nih.gov/Blast.cgi ) using the accession numbers XP_001488401.2 (Equus caballus) and NP_034063.1 (Mus musculus). (DOC) [file pone.0105964.s001.doc]

Subject (sbjct): **collagen alpha-1 VI chain [Equus caballus]**

XP_001488401.2

Query: **collagen alpha-1 VI chain [Mus musculus]**

NP_034063.1

Score: 1665 bits(4312) Method: Compositional matrix adjust.

Identities: 896/1006(89%) Positives 941/1006(93%) Gaps: 1/1006(0%)

Features:

Query 21 DIQGSKAIAFQDCPVDLFFVLDTSESVALRLKPYGALVDKVKSFTKRFIDNLRDRYYRCD 80

D + +AFQDCPVDLFFVLDTSESVALRLKPYGALVDKVK+FTKRFIDNLRDRYYRCD

Sbjct 22 DTAAVRTVAFQDCPVDLFFVLDTSESVALRLKPYGALVDKVKAFTKRFIDNLRDRYYRCD 81

Query 81 RNLVWNAGALHYSDEVEIIRGLTRMPSGRDELKASVDAVKYFGKGTYTDCAIKKGLEELL 140

RNLVWNAGALHYSDEVEIIRGLTRMPSGRDELKASVDAVKYFGKGTYTDCAIKKGLEELL

Sbjct 82 RNLVWNAGALHYSDEVEIIRGLTRMPSGRDELKASVDAVKYFGKGTYTDCAIKKGLEELL 141

Query 141 IGGSHLKENKYLIVVTDGHPLEGYKEPCGGLEDAVNEAKHLGIKVFSVAITPDHLEPRLS 200

+GGSHLKENKYLIVVTDGHPLEGYKEPCGGLEDAVNEAKHLGIKVFSVAITPDHLEPRLS

Sbjct 142 VGGSHLKENKYLIVVTDGHPLEGYKEPCGGLEDAVNEAKHLGIKVFSVAITPDHLEPRLS 201

Query 201 IIATDHTYRRNFTAADWGHSRDAEEVISQTIDTIVDMIKNNVEQVCCSFECQAARGPPGP 260

IIATDHTYRRNFTAADWG SRDAEE+ISQTIDTI DMIKNNVEQVCCSFECQ ARGPPG

Sbjct 202 IIATDHTYRRNFTAADWGQSRDAEEIISQTIDTITDMIKNNVEQVCCSFECQPARGPPGL 261

Query 261 RGDPGYEGERGKPGLPGEKGEAGDPGRPGDLGPVGYQGMKGEKGSRGEKGSRGPKGYKGE 320

RGDPGYEGERGKPGLPGEKGEAGDPGRPGDLGPVGYQGMKGEKGSRG+KGSRGPKGYKGE

Sbjct 262 RGDPGYEGERGKPGLPGEKGEAGDPGRPGDLGPVGYQGMKGEKGSRGDKGSRGPKGYKGE 321

Query 321 KGKRGIDGVDGMKGETGYPGLPGCKGSPGFDGIQGPPGPKGDAGAFGMKGEKGEAGADGE 380

KGKRGIDGVDGMKGETGYPGLPGCKGSPG DG+QGPPGPKGDAGAFG+KG KGE GADGE

Sbjct 322 KGKRGIDGVDGMKGETGYPGLPGCKGSPGLDGVQGPPGPKGDAGAFGLKGAKGEPGADGE 381

Query 381 AGRPGNSGSPGDEGDPGEPGPPGEKGEAGDEGNAGPDGAPGERGGPGERGPRGTPGVRGP 440

GRPG++G PGDEG+PGEPGPPGEKGEAGDEGN+GPDG PG+RGGPGERGPRGTPGVRGP

Sbjct 382 PGRPGDTGPPGDEGEPGEPGPPGEKGEAGDEGNSGPDGPPGDRGGPGERGPRGTPGVRGP 441

Query 441 RGDPGEAGPQGDQGREGPVGIPGDSGEAGPIGPKGYRGDEGPPGPEGLRGAPGPVGPPGD 500

RGDPGEAGPQGDQGREGPVG+PGD GEAGPIGPKGYRGDEGPPG EG RGAPGP GPPGD

Sbjct 442 RGDPGEAGPQGDQGREGPVGVPGDPGEAGPIGPKGYRGDEGPPGLEGPRGAPGPAGPPGD 501

Query 501 PGLMGERGEDGPPGNGTEGFPGFPGYPGNRGPPGLNGTKGYPGLKGDEGEVGDPGEDNND 560

PGLMG RGEDGPPGNGTEGFPGFPGYPG+RGPPG+NGTKGYPGLKGDEGE GDPGEDN D

Sbjct 502 PGLMGARGEDGPPGNGTEGFPGFPGYPGSRGPPGINGTKGYPGLKGDEGEAGDPGEDNTD 561

Query 561 ISPRGVKGAKGYRGPEGPQGPPGHVGPPGPDECEILDIIMKMCSCCECTCGPIDILFVLD 620

ISPRGVKGAKGYRGPEGP GPPG G P DECEILDIIMKMCSCCEC CGPIDILFVLD

Sbjct 562 ISPRGVKGAKGYRGPEGPPGPPGPPGRPEMDECEILDIIMKMCSCCECKCGPIDILFVLD 621

Query 621 SSESIGLQNFEIAKDFIIKVIDRLSKDELVKFEPGQSHAGVVQYSHNQMQEHVDMRSPNV 680

SSESIGLQNFEIAKDFI+KVIDRLS+DELVKFE GQSHAGVVQYSHNQMQEHV + PN+

Sbjct 622 SSESIGLQNFEIAKDFIVKVIDRLSRDELVKFEAGQSHAGVVQYSHNQMQEHVGLSDPNI 681

Query 681 RNAQDFKEAVKKLQWMAGGTFTGEALQYTRDRLLPPTQNNRIALVITDGRSDTQRDTTPL 740

RN Q+ KEA+KKLQWMAGGTFTGEALQYTR RLLPPTQN RIALVITDGRSDTQRDTTPL

Sbjct 682 RNTQELKEAIKKLQWMAGGTFTGEALQYTRSRLLPPTQNTRIALVITDGRSDTQRDTTPL 741

Query 741 SVLCGADIQVVSVGIKDVFGFVAGSDQLNVISCQGLS-QGRPGISLVKENYAELLDDGFL 799

SVLCG DIQVVSVGIKDVFG +AGSDQLNVISCQGL+ QGRPGISLVKENYAELL+D FL

Sbjct 742 SVLCGPDIQVVSVGIKDVFGSIAGSDQLNVISCQGLAPQGRPGISLVKENYAELLEDAFL 801

Query 800 KNITAQICIDKKCPDYTCPITFSSPADITILLDSSASVGSHNFETTKVFAKRLAERFLSA 859

KNIT QICIDKKCPDYTCPITFSSPADITILLD SASVGSHNF+ TK FAKRLAERFL+A

Sbjct 802 KNITTQICIDKKCPDYTCPITFSSPADITILLDGSASVGSHNFDITKRFAKRLAERFLTA 861

Query 860 GRADPSQDVRVAVVQYSGQGQQQPGRAALQFLQNYTVLASSVDSMDFINDATDVNDALSY 919

R +P+ +VRV+V+QYSG GQQQP RA+LQFLQNYTVLAS++DSMDFINDATDV DALSY

Sbjct 862 RRKNPAHEVRVSVMQYSGTGQQQPERASLQFLQNYTVLASTIDSMDFINDATDVTDALSY 921

Query 920 VTRFYREASSGATKKRVLLFSDGNSQGATAEAIEKAVQEAQRAGIEIFVVVVGPQVNEPH 979

VTRFYREASS +R+LLFSDGNSQGATA AI+KAVQEAQRA IE+FVVVVG VNEPH

Sbjct 922 VTRFYREASSEEVDRRLLLFSDGNSQGATAAAIKKAVQEAQRANIEVFVVVVGTHVNEPH 981

Query 980 IRVLVTGKTAEYDVAFGERHLFRVPNYQALLRGVLYQTVSRKVALG 1025

IRVLVT KTAEY+VA+G+RH FRV +YQ LL GV YQ+VSRKVA+G

Sbjct 982 IRVLVTDKTAEYNVAYGQRHQFRVASYQELLGGVFYQSVSRKVAMG 1027
