# Supplementary material for: Immunogenicity of Intensively Decellularized Equine Carotid Arteries Is Conferred by the Extracellular Matrix Protein Collagen Type VI
Source: PLoS One. 2014 Aug 26;9(8):e105964. doi: 10.1371/journal.pone.0105964 (PMC4144968; doi:10.1371/journal.pone.0105964)
Supplement: Figure S2 — Alignment of equine and murine collagen alpha-2 VI amino acid sequences by basic local alignment search tool (BLAST, http://blast.ncbi.nlm.nih.gov/Blast.cgi ) using the accession numbers XP_005606307.1 (Equus caballus) and AAH34414 (Mus musculus). (DOC) [file pone.0105964.s002.doc]

Subject (Sbjct): **collagen alpha-2 VI chain [Equus caballus]**

XP_005606307.1

Query: **collagen alpha-2 VI chain [Mus musculus]**

AAH34414

Score: 1665 bits(4312) Method: Compositional matrix adjust.

Identities: 911/982(93%) Positives: 944/982(96%) Gaps: 1/982(0%)

Features :

Query 53 PEKADCPVNVYFVLDTSESVAMQSPTDSLLYHMQQFVPQFISQLQNEFYLDQVALSWRYG 112

PEKADCPVNVYFVLDTSESV MQSPTDSLLYHMQQFVPQFISQLQ+EFYLDQVALSWRYG

Sbjct 237 PEKADCPVNVYFVLDTSESVTMQSPTDSLLYHMQQFVPQFISQLQDEFYLDQVALSWRYG 296

Query 113 GLHFSDQVEVFSPPGSDRASFTKSLQGIRSFRRGTFTDCALANMTQQIRQHVGKGVVNFA 172

GLHFSDQVEVFSPP SDRASF KSLQ I SFRRGTFTDCALANMTQ+IR+HV KGV NFA

Sbjct 297 GLHFSDQVEVFSPPNSDRASFIKSLQSISSFRRGTFTDCALANMTQEIRRHVKKGV-NFA 355

Query 173 VVITDGHVTGSPCGGIKMQAERAREEGIRLFAVAPNRNLNEQGLRDIANSPHELYRNNYA 232

VVITDGHVTGSPCGGIK QAERAREEGIRLFAVAPNRNL+EQGLRDIA++P ELYR+NYA

Sbjct 356 VVITDGHVTGSPCGGIKRQAERAREEGIRLFAVAPNRNLHEQGLRDIASTPLELYRSNYA 415

Query 233 TMRPDSTEIDQDTINRIIKVMKHEAYGECYKVSCLEIPGPHGPKGYRGQKGAKGNMGEPG 292

TMRPDST+IDQDTINRIIKVMKHEAYGECYKVSCLEIPGP GPKGYRGQKGAKGNMGEPG

Sbjct 416 TMRPDSTDIDQDTINRIIKVMKHEAYGECYKVSCLEIPGPPGPKGYRGQKGAKGNMGEPG 475

Query 293 EPGQKGRQGDPGIEGPIGFPGPKGVPGFKGEKGEFGSDGRKGAPGLAGKNGTDGQKGKLG 352

EPGQKGRQGDPGIEGPIGFPGPKGVPGFKGEKGEFG+DGRKGAPGLAGKNGTDGQKGKLG

Sbjct 476 EPGQKGRQGDPGIEGPIGFPGPKGVPGFKGEKGEFGADGRKGAPGLAGKNGTDGQKGKLG 535

Query 353 RIGPPGCKGDPGSRGPDGYPGEAGSPGERGDQGAKGDSGRPGRRGPPGDPGDKGSKGYQG 412

RIGPPGCKGDPG+RGPDGY GEAGSPGE+GDQGAKGD GRPGRRGPPG+ G KGSKGYQG

Sbjct 536 RIGPPGCKGDPGNRGPDGYVGEAGSPGEQGDQGAKGDPGRPGRRGPPGEDGAKGSKGYQG 595

Query 413 NNGAPGSPGVKGGKGGPGPRGPKGEPGRRGDPGTKGGPGSDGPKGEKGDPGPEGPRGLAG 472

NNG+PGSPGVKG KGGPGPRGPKGEPGRRGDPGTKGGPGSDGPKGEKGDPGPEGPRGLAG

Sbjct 596 NNGSPGSPGVKGAKGGPGPRGPKGEPGRRGDPGTKGGPGSDGPKGEKGDPGPEGPRGLAG 655

Query 473 EVGSKGAKGDRGLPGPRGPQGALGEPGKQGSRGDPGDAGPRGDSGQPGPKGDPGRPGFSY 532

EVG+KGAKGDRGLPGPRGPQGALGEPGKQGSRGDPGDAGPRGDSGQPGPKGDPGRPGFSY

Sbjct 656 EVGNKGAKGDRGLPGPRGPQGALGEPGKQGSRGDPGDAGPRGDSGQPGPKGDPGRPGFSY 715

Query 533 PGPRGTPGEKGEPGPPGPEGGRGDFGLKGTPGRKGDKGEPADPGPPGEPGPRGPRGIPGP 592

PGPRG PGEKGEPGP GPEGGRGDFG+KG PGRKG KGEPADPGPPGEPGPRGPRG PG

Sbjct 716 PGPRGEPGEKGEPGPRGPEGGRGDFGMKGAPGRKGQKGEPADPGPPGEPGPRGPRGEPGL 775

Query 593 EGEPGPPGDPGLTECDVMTYVRETCGCCDCEKRCGALDVVFVIDSSESIGYTNFTLEKNF 652

EGEPGPPGDPGLTECDVMTYVRETCGCCDCEKRCGALDVVFVIDSSESIGYTNFTLEKNF

Sbjct 776 EGEPGPPGDPGLTECDVMTYVRETCGCCDCEKRCGALDVVFVIDSSESIGYTNFTLEKNF 835

Query 653 VINVVNRLGAIAKDPKSETGTRVGVVQYSHEGTFEAIRLDDERVNSLSSFKEAVKNLEWI 712

VINVVNRLGAIAKDPKSETGTRVGVVQYSHEGTFEAI+LDDER+NSLSSFKEAVKNLEWI

Sbjct 836 VINVVNRLGAIAKDPKSETGTRVGVVQYSHEGTFEAIQLDDERINSLSSFKEAVKNLEWI 895

Query 713 AGGTWTPSALKFAYNQLIKESRRQKTRVFAVVITDGRHDPRDDDLNLRALCDRDVTVTAI 772

AGGTWTPSALKFAYNQLIKESRRQKTRVFAVVITDGRHDPRDDDLNLRALC+ DVTVTAI

Sbjct 896 AGGTWTPSALKFAYNQLIKESRRQKTRVFAVVITDGRHDPRDDDLNLRALCNHDVTVTAI 955

Query 773 GIGDMFHETHESENLYSIACDKPQQVRNMTLFSDLVAEKFIDDMEDVLCPDPQIVCPELP 832

GIGDMFHE HESENLYSIACDKPQQVRNMTLFSDLVAEKFIDDMEDVLCPDPQIVCP+LP

Sbjct 956 GIGDMFHERHESENLYSIACDKPQQVRNMTLFSDLVAEKFIDDMEDVLCPDPQIVCPDLP 1015

Query 833 CQTELYVAQCTQRPVDIVFLLDGSERLGEQNFHKVRRFVEDVSRRLTLARRDDDPLNARM 892

CQTELYVAQCTQRPVDIVFLLDGSERLGEQNFHK RRFVE+VSRRLTLARR+DDPLNAR+

Sbjct 1016 CQTELYVAQCTQRPVDIVFLLDGSERLGEQNFHKARRFVEEVSRRLTLARREDDPLNARV 1075

Query 893 ALLQYGSQNQQQVAFPLTYNVTTIHEALERATYLNSFSHVGTGIVHAINNVVRGARGGAR 952

ALLQ+G +++QQVAFPLT N+T IHEAL A YLNSFSHVG GIVHAIN VV+ AR GAR

Sbjct 1076 ALLQFGGRDEQQVAFPLTSNLTAIHEALASARYLNSFSHVGAGIVHAINYVVQDARAGAR 1135

Query 953 RHAELSFVFLTDGVTGNDSLEESVHSMRKQNVVPTVVAVGGDVDMDVLTKISLGDRAAIF 1012

RHAEL+FVFLTDGVTGNDSLEE+VHSMRKQNVVPTVVAVG DVD DVL+KISLGD AA+F

Sbjct 1136 RHAELAFVFLTDGVTGNDSLEEAVHSMRKQNVVPTVVAVGSDVDADVLSKISLGDAAAVF 1195

Query 1013 REKDFDSLAQPSFFDRFIRWIC 1034

REKD+DSLAQP FFDRFIRWIC

Sbjct 1196 REKDYDSLAQPGFFDRFIRWIC 1217
